# Stimulation of hair regrowth in an animal model of androgenic alopecia using 2-deoxy-D-ribose

**DOI:** 10.3389/fphar.2024.1370833

**Published:** 2024-06-03

**Authors:** Muhammad Awais Anjum, Saima Zulfiqar, Aqif Anwar Chaudhary, Ihtesham Ur Rehman, Anthony J. Bullock, Muhammad Yar, Sheila MacNeil

**Affiliations:** ^1^ Interdisciplinary Research Center in Biomedical Materials, COMSATS University Islamabad, Lahore Campus, Lahore, Pakistan; ^2^ School of Medicine, University of Central Lancashire, Preston, United Kingdom; ^3^ Department of Materials Science and Engineering, Kroto Research Institute, University of Sheffield, Sheffield, United Kingdom

**Keywords:** androgenic alopecia, 2-deoxy-D-ribose, C57BL6 mice, testosterone, minoxidil, hair regrowth, chemotherapy

## Abstract

Androgenic alopecia (AGA) affects both men and women worldwide. New blood vessel formation can restore blood supply and stimulate the hair regrowth cycle. Recently, our group reported that 2-deoxy-D-ribose (2dDR) is 80%–90% as effective as VEGF in the stimulation of neovascularization in *in vitro* models and in a chick bioassay. In this study, we aimed to assess the effect of 2dDR on hair growth. We prepared an alginate gel containing 2dDR, polypropylene glycol, and phenoxyethanol. AGA was developed in C57BL6 mice by intraperitoneally injecting testosterone (TE). A dihydrotestosterone (DHT)-treated group was used as a negative control, a minoxidil group was used as a positive control, and we included groups treated with 2dDR gel and a combination of 2dDR and minoxidil. Each treatment was applied for 20 days. Both groups treated with 2dDR gel and minoxidil stimulated the morphogenesis of hair follicles. H&E-stained skin sections of C57BL/6 mice demonstrated an increase in length, diameter, hair follicle density, anagen/telogen ratio, diameter of hair follicles, area of the hair bulb covered in melanin, and an increase in the number of blood vessels. Masson’s trichrome staining showed an increase in the area of the hair bulb covered in melanin. The effects of the FDA-approved drug (minoxidil) on hair growth were similar to those of 2dDR (80%–90%). No significant benefit were observed by applying a combination of minoxidil with 2dDR. We conclude that 2dDR gel has potential for the treatment of androgenic alopecia and possibly other alopecia conditions where stimulation of hair regrowth is desirable, such as after chemotherapy. The mechanism of activity of 2dDR remains to be established.

## 1 Introduction

Alopecia can occur due to hormonal imbalance, thyroid problems, certain medications, and autoimmune diseases. It can be induced by blood thinning medications, contraceptives, antidepressants, steroidal anti-inflammatory drugs, beta and calcium-channel blockers, retinoids, and chemotherapeutics (Vicky et al., 2018). Male pattern baldness, also known as androgenic alopecia (AGA), is one of the most widespread hair loss conditions in the world (Yohei et al., 2018). In the pathophysiology of AGA, testosterone is converted to dihydrotestosterone (DHT) by 5α-reductase. DHT then binds to androgen receptors in the dermal papilla cells (DPCs) of sensitive hair follicles and prolongs the telogen phase, causing hair loss before the growth of new hair (Izabela et al., 2014). AGA is said to affect 30% of Asian men by age 30 and 50% by age 50 (Yohei et al., 2018). It also affects 80% of White men and 40% of White women by age 70 (Pietro et al., 2019). Platelet-rich plasma (PRP) injections, topical drugs, oral medications, and hair transplant operations are currently being used to treat AGA (Alfredo et al., 2016). Currently, minoxidil and finasteride are the only FDA-approved drugs used to treat AGA. Minoxidil facilitates hair growth by facilitating DP survival and expanding the anagenic phase (Hyun et al., 2004). Finasteride stimulates the growth of new hair by suppressing the activity of 5α-reductase (Libecco and Bergfeld, 2004). Both medications have side effects; for example, finasteride has been reported to reduce sexual drive, while minoxidil can trigger acute anteroseptal infarction, myocardial infarction, and anorexia (Hiroshi et al., 2000; Vesoulis et al., 2014).

Minoxidil usually causes early shedding of hairs that are already in the telogen phase (telogen effluvium) because of shortening of the telogen phase before refreshing the growth of healthy hair. This requires long-term application of minoxidil for sustained effect (Alfredo et al., 2012). The common side effects of topical minoxidil are dermatological, such as contact dermatitis, pruritus, and erythema. To date, there is only one case report of heart failure with use of topical minoxidil (Hiroshi et al., 2000; Friedman et al., 2002). Many intracellular signaling molecules encourage the proliferation of dermal papillary cells—including kinases and growth factors—and hence play an essential part in stimulating hair growth. The signaling protein vascular endothelial growth factor (VEGF) is secreted from the vascular epithelium and stimulates angiogenesis through intracellular pathways. This extends the vascular network surrounding the hair follicle, supporting hair regrowth (Mauro et al., 2001; Choi, 2018). Shin et al. demonstrated that Rg3 (one of the major components in *Panax ginseng*) stimulated VEGF mRNA levels while also increasing the proliferation of human dermal papillary cells. Rg3 activated stem cells in mouse hair follicles *in vivo* by upregulating factor-activating CD34 and was found to promote hair growth better than minoxidil (Hyun et al., 2014). Theoretically, any agents that can stimulate the production of VEGF could be useful in the regeneration of hair in the telogen phase.

2-Deoxy-D-ribose (2dDR) is a D-isomer of a deoxypentose monosaccharide, in which a hydrogen atom is present with a hydroxyl group at the C-2 position, in place of the hydroxyl group. 2dDR is known to enhance tubulogenesis (Ikeda et al., 2006), prevent hypoxia-induced apoptosis (Yuichi et al., 2004), and boost VEGF and IL-8 production (Serkan et al., 2021) of ECs *in vitro*, which is consistent with the stimulatory effects of 2dDR on cell proliferation and migration. Our group has found that 2dDR stimulated angiogenesis, proliferation of endothelial cells, and accelerated wound healing in rat models (Yar et al., 2017). These studies (Yar et al., 2017; Serkan et al., 2019; Anisa et al., 2020; Serkan et al., 2020; Serkan et al., 2021) collectively make a substantial contribution to the understanding of the biological (pro-angiogenic) activity of 2dDR. Additionally, we have demonstrated that 2dDR can be loaded into several biomaterials for prolonged release over several days to promote the growth of neonatal blood vessels (Yar et al., 2017; Maryam et al., 2019; Serkan et al., 2021). Sodium alginate, a biodegradable, non-toxic, and water-soluble macromolecule, is an ideal polymer for gels (Wang and Wang, 2010). Propylene glycol (PG) is a viscous liquid used in pharmacological delivery systems for its hydrophilic penetration and antiseptic properties (Mujica et al., 2016). It improves fluid retention time in hydrogels and interacts with intercellular lipids for stratum corneum barrier penetration (Trommer and Neubert, 2006; Victor et al., 2020). Phenoxyethanol is an approved stabilizer and antimicrobial agent, preventing contamination in hydrogels (Nolan and Nolan, 1972).

Our aim in the current study was to investigate whether delivery of 2dDR from hydrogels would stimulate hair regeneration via angiogenesis. For this study, we prepared hydrogels comprising sodium alginate and propylene glycol, with phenoxyethanol added as a stabilizer (blank-SA). It was found that 2dDR-SA hydrogels, when supplemented with 2dDR, showed sustained hair growth in AGA mice, demonstrating 2dDR-SA hydrogel as a potential therapeutic agent for treating AGA.

## 2 Materials and methods

### 2.1 Materials

Sodium alginate (Cat. No. 7528-1405) with a molecular weight of 120,000–190,000 g/moL was purchased from Daejung Chemicals, Korea. Propylene glycol (99.5% pure; Cat. No. 24300-11000PE) was purchased from Penta Chemicals Limited, Czech Republic. 2-Phenoxyethanol (94% pure; Cat. No. A10786.30) was purchased from Alfa Aesar, United States. 2-Deoxy-D-ribose sugar (Cat. No. 00613) was purchased from Chem-Impex International, United States. Minoxidil (Hair Max^®^ 2%) was purchased from Sante (Private) Limited, Karachi, Pakistan, and testosterone enanthate (brand name Testoviron Depot^®^) was purchased from BAYER Pharmaceuticals, Germany. Autoclaved deionized water was used in the manufacturing process in the current research. All chemicals and reagents were of analytical grade and used without any further purification.

### 2.2 Preparation of control and2dDR-loaded hydrogels

Two different hydrogels, blank-SA and 2dDR-SA, were prepared by simple manual mixing of the constituents in autoclaved sterilized water at RT by using a spatula. The blank-SA hydrogel was composed of 1.4 g of sodium alginate (6.4% w/w), 250 mg of propylene glycol (1.15% w/w), and 82.5 mg of 2-phenoxyethanol (0.377% w/w) as a stabilizer in 20 mL water. The 2dDR-SA hydrogel was composed of 1.4 g sodium alginate (6.416% w/w), 250 mg propylene glycol (1.146% w/w), 82.5 mg of 2-phenoxyethanol (0.375% w/w), and 86.62 mg of 2-deoxy-D-ribose sugar (0.394% w/w) in 20 mL water. The prepared hydrogels (blank-SA and 2dDR-SA) were stored in glass vials at RT.

For FTIR and 2dDR release studies, 10 mL each of blank-SA and 2dDR-SA hydrogels were poured into Petri dishes (100 × 15 mm) and covered with a perforated aluminum foil. These were frozen at −20°C for 20 h and then placed in Labconco’s FreeZone (4.5 L) at −105°C for 24 h. These freeze-dried hydrogels (FD-blank-SA and FD-2dDR-SA) were stored at RT until used for analyses.

### 2.3 Fourier-transform infrared spectroscopy (FTIR) analysis

The FTIR technique is used to estimate a sample’s interference pattern of emission or absorption. Chemical characteristics of manufactured sodium alginate-based 2dDR hydrogel and sodium alginate Blank-Gel were observed on a spectroscopic analysis (FTIR) system in the frequency range of 4,000–400 cm^−1^ with 256 sequential scans at 8 cm^−1^ rulings on a photo audio mode, which is a way to examine materials with no specimen preparation (Thermo Nicolet 6700P, United States spectrometer).

### 2.4 Analysis of 2dDR release from the 2dDR-SA hydrogel

2dDR release from the 2dDR-SA hydrogel was quantitatively determined by Dische’s diphenylamine assay (Jennifer and Mura, 2013). A stock solution of Dische’s reagent was prepared by dissolving 0.75 g diphenylamine in a solution of 50 mL glacial acetic acid supplemented with 750 µL of concentrated sulfuric acid in dark conditions. A fresh solution of 2% v/v ethanol in distilled water was prepared for the assay, and 50 µL of the ethanol solution was added to 10 mL of the diphenylamine solution.

Room-temperature dried 2dDR-SA hydrogel was cut into 2 × 2 cm patches and immersed in PBS in a six-well plate in a sterile environment. The plate was tightly sealed with Parafilm in order to avoid evaporation of PBS and incubated at 37°C and 55% humidity. The release of 2dDR was monitored at different time points (4 h and 1 d, 2 d, 3 d, 4 d, and 7 d). At each time point, the release medium (PBS) from each well was extracted and replaced with fresh media. Dische’s reagent (500 µL) was added to 500 µL of release media and incubated for 10 min at 100°C. Samples were then allowed to cool to room temperature for 10 min before centrifugation at 10,000 rpm for 5 min. The absorbance of the resulting supernatant at 590 nm was measured. The concentration of the released 2dDR was obtained from a calibration curve of known dilutions of 2dDR. Cumulative drug release vs. time was calculated.

### 2.5 Development of the androgenic alopecia model in male C57BL/6 mice and testing of blank-SA and 2dDR-SA hydrogels against androgenic alopecia

Seven-week-old male C57BL/6 mice were purchased from the Center of Excellence in Molecular Biology, Punjab University Lahore, Pakistan. The mice were housed in plastic cages at ambient temperature (25°C ± 2°C) under controlled light/dark cycles of 12 h/12 h and fed standard mouse chow and water *ad libitum*. They were acclimated to the laboratory environment for 1 week. All the principles of laboratory animal care were followed at the Pre-Clinical Research Facility, IRCBM, CUI, Lahore, and all animal experiments were carried out following the Guidelines for the Care and Use of Laboratory Animals by CUI, Lahore Ethical Committee with Ethical approval certificate number EC/MY/007/22.

In brief, animals were randomly divided into six groups of three mice in each NC and T-1, while four in all other four treatment groups. Treatment groups were referred to as T1–T5, as described in Table 1. The androgenic alopecia model was developed in C57BL/6 mice by intraperitoneally injecting testosterone enanthate at 0.1 mL of 5 mg/mL (20 mg/kg) three times per week for 2 weeks, as previously described in the literature, with slight modifications (Fu et al., 2021). After 2 weeks, hair on the treated back skin (2 × 3 cm) of mice were depilated using a hair removing cream under general anesthesia using ketamine at 80 mg/kg I/P and xylazine at 10 mg@/kg I/P. Approximately, 0.5 mL of each hydrogel (blank-SA and 2dDR-SA) was applied topically on the dorsal side of mice (2 × 3 cm) once daily for 20 days, and the experiment was terminated on day 21. Digital photographs were taken on days 0, 7, 14, and 21 using a DSLR camera. Animals were sacrificed by cervical dislocation before hair and tissue samples were collected.

**TABLE 1:** Different treatment groups of C57BL/6 mice subjected for hair regrowth evaluation.

Sr. no.	Groups	Androgenic alopecia (inj. of testosterone)	Treatment	No. of mice
1.	NC (normal control)	No	No	3
2.	T-1 (model group)	Yes	No	3
3.	T-2 (blank-SA)	Yes	Blank-SA	4
4.	T-3 (2dDR-SA)	Yes	2dDR-SA	4
5.	T-4 (positive group)	Yes	Minoxidil 2% spray	4
6.	T-5 (mixed group)	Yes	2dDR + Minoxidil	4

The treatment groups in experimental animals are given in Table 1.

### 2.6 Hair shaft and hair length analysis

On day 21, at least 70 hairs per mouse were collected from three mice in each group, as described (Valerie et al., 2001). There are usually four types of hair present in the scalp of C57BL6 mice, which are described as guard, awl, auchene, and zigzag. Large guard hairs are first formed during the embryogenesis of hair. We selected 20 guard hairs from 70 random hair samples. The guard hairs were distinguished mainly by their length and the diameter of the hair shaft or thickness of hair. The length of hair was measured by using a digital vernier caliper, while the hair shaft analysis of guard type hairs was done by taking pictures of the hair shaft portions using an inverted microscope (Optika, Italy) at ×40 magnification.

### 2.7 Histological evaluation

To evaluate skin morphology and hair regrowth, 2 × 3 cm skin samples were collected on day 21 (after depilation). Samples were fixed in 4% paraformaldehyde for 24 h and then embedded in paraffin blocks. Longitudinal and horizontal sections of 5.0-μm-thick skin were prepared and stained with hematoxylin and eosin (H&E). Photographs of H&E-stained sections were taken using an inverted microscope (Optika, Italy). The longitudinal sections were stained using Masson’s trichrome staining to evaluate the area covered by melanin in the hair bulb. The sections were evaluated for follicular length, follicular diameters, and follicular density. The horizontal sections were evaluated for anagen follicle (A) and telogen follicle (T), and the A/T ratio was calculated (Ying et al., 2017) using an inverted microscope (Optika, Italy), and images were captured. Four parameters, 1) number of hair follicles; 2) hair bulb diameter; 3) follicular density (number of hair follicles per mm^2^); and 4) number of anagen (A) and telogen (T) follicles per millimeter area, were measured to evaluate hair growth using ImageJ software (NIH, MD, United States). To assess the angiogenesis potential of 2dDR-SA hydrogels in promoting hair growth in AGA-induced mice, blood vessels were counted in cross-sections of H&E staining.

### 2.8 Statistical analysis

All experiments were conducted in triplicate or more, and their results were calculated as mean ± S.D. Statistical analysis (unpaired Student’s t-test) was performed via GraphPad QuickCalcs (https://www.graphpad.com/quickcalcs/ttest1.cfm). All results with a *p*-value ≤ 0.05 were considered statistically significant.

## 3 Results

This study focused on the development of a 2dDR-releasing sodium alginate-based tube hydrogel to promote the growth of hair in AGA-induced mouse model. Hydrogels were synthesized by the simple mixing of sodium alginate and propylene glycol with 2-phenoxyethanol in water with and without 2dDR. These were freeze-dried for FTIR and release studies of 2dDR, while for *in vivo* studies, wet hydrogels were used. The results of the FTIR and release studies of 2dDR are described in the following sections.

### 3.1 FTIR of blank-SA and 2dDR-SA hydrogels

**FIGURE 1:**
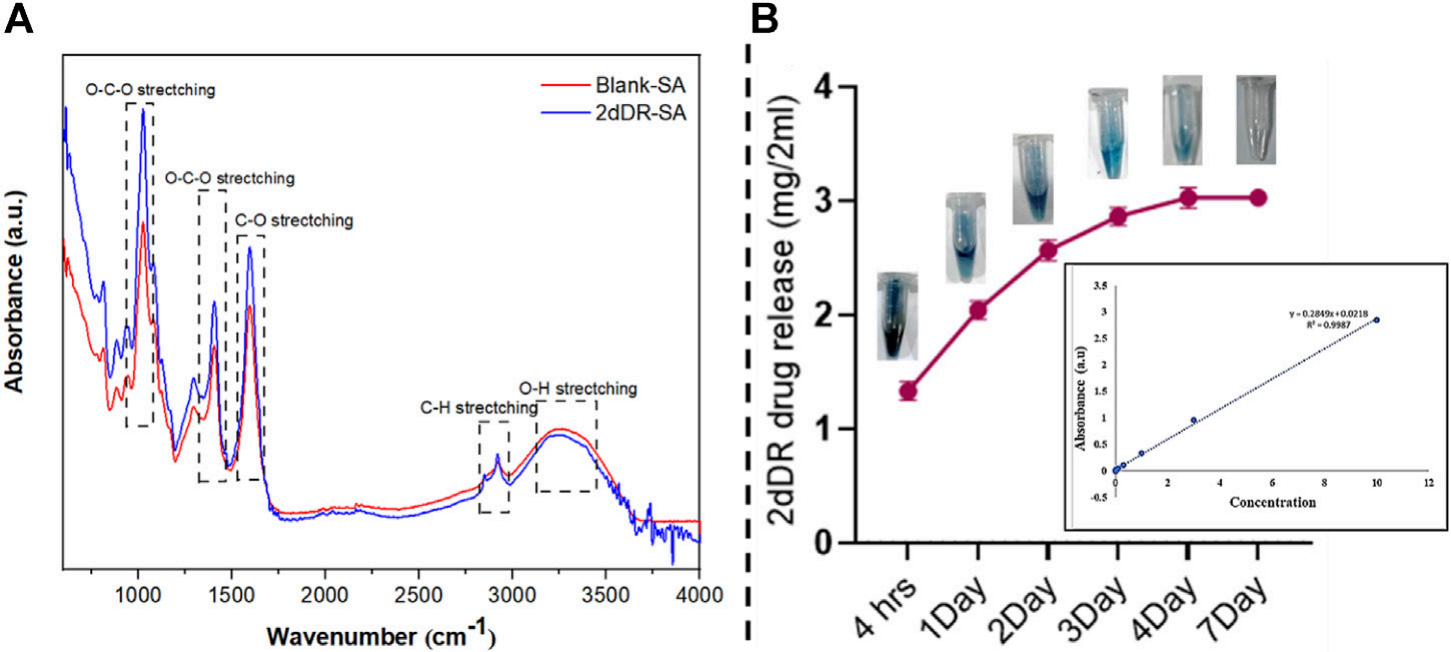
**(A)** FTIR spectra of blank-SA and 2dDR-SA hydrogels; **(B)** release and colorimetric detection of 2dDR at different time intervals (4 h and 1, 2, 3, 4, and 7 days) of drug release analysis.

FTIR analysis was used to determine the essential structural and chemical properties of the blank-SA and 2dDR-SA hydrogels and validate the formation of a hydrogel matrix. This method also records structural changes caused by any effects of the drug on the carrier hydrogel. The results of the FTIR spectra of the hydrogels shown in Figure 3A were drawn using Origin software (OriginLab, United States) and cross-checked against literature. The spectra obtained for 2dDR, blank-SA, and 2dDR-SA are presented in Figure 4. Sodium alginate exhibited absorption band characteristics at 3,410 cm^−1^, which can be due to hydroxyl group (–OH) (Thaned, 2009; Jing et al., 2010); 1,635 cm^−1^ due to the asymmetric stretching vibration of COO groups; 1,419 cm^−1^ due to the symmetric stretching vibration of COO groups; 1,050 cm^−1^ due to the elongation of C–O groups; and 1,294 cm^−1^ and 1,024 cm^−1^ due to C–O and C–O–C stretching vibrations of alcoholic hydrogen bonding, respectively (Rúben et al., 2011). Bands at 2,859 cm^−1^ and 2,970 cm^−1^ corresponded to C–H stretching, and the bands at 3,410 cm^−1^ corresponded to the hydroxyl group of propylene glycol (Munirah et al., 2016). Characteristic intense bands associated with O–C, C–C, and C–O stretching modes were observed at 1,078 and 1,042 cm^−1^. The very intense Raman spectra at 996 and 1,006 cm^−1^ were the characteristic ring band of 2-phenoxyethanol. The observed intense bands at 750 and 688 cm^−1^ were assigned to the C–H ring wagging modes of 2-phenoxyethanol (Badawi, 2011). In comparison to the peaks of scaffolds without 2dDR, FTIR spectra show that 5% 2dDR-loaded hydrogel has no peak shift. Thus, 2dDR has no effect on the sodium-alginate structure (Figure 1A).

### 3.2 2dDR release analysis from 2dDR-SA hydrogel

Developing hydrogels with controlled release of drugs is desirable for wound healing applications. The 2dDR drug release was measured using Dische’s diphenylamine assay. This assay is based on 2dDR being changed by the acidic environment into an aldehyde molecule, which then interacts with diphenylamine to form a blue complex that can be easily measured using UV/Vis absorption spectroscopy. For this purpose, the 2dDR release from room temperature-dried 2dDR-SA was performed in PBS at 37°C for different time intervals (4, 24, 48, 72, 96, and 168 h), and the results are shown in Figure 1B. After 4 h, the 2dDR-SA hydrogel released 70.3% (1.33 mg/2 mL) of the total drug. The release was 79.4% (2.045 mg/2 mL) by day 1; by day 2, 84% release (2.60 mg/2 mL); by day 3, 87% release (2.88 mg/2 mL); and by day 4, 89% release [3.50 mg/2 (Figure1B)]. The 2dDR-SA displayed the same release of 89% (3.50 mg/2 mL) on day 7 as it did on the 4^th^ day, and this was the release of essentially all of the 2dDR from the hydrogel.

This shows that 2dDR is slowly released at 37°C for up to 96 h, thus reducing the need for repeated treatments on a daily basis.

### 3.3 Promoting regrowth of hair in the testosterone-induced androgenic alopecia mouse model

**FIGURE 2:**
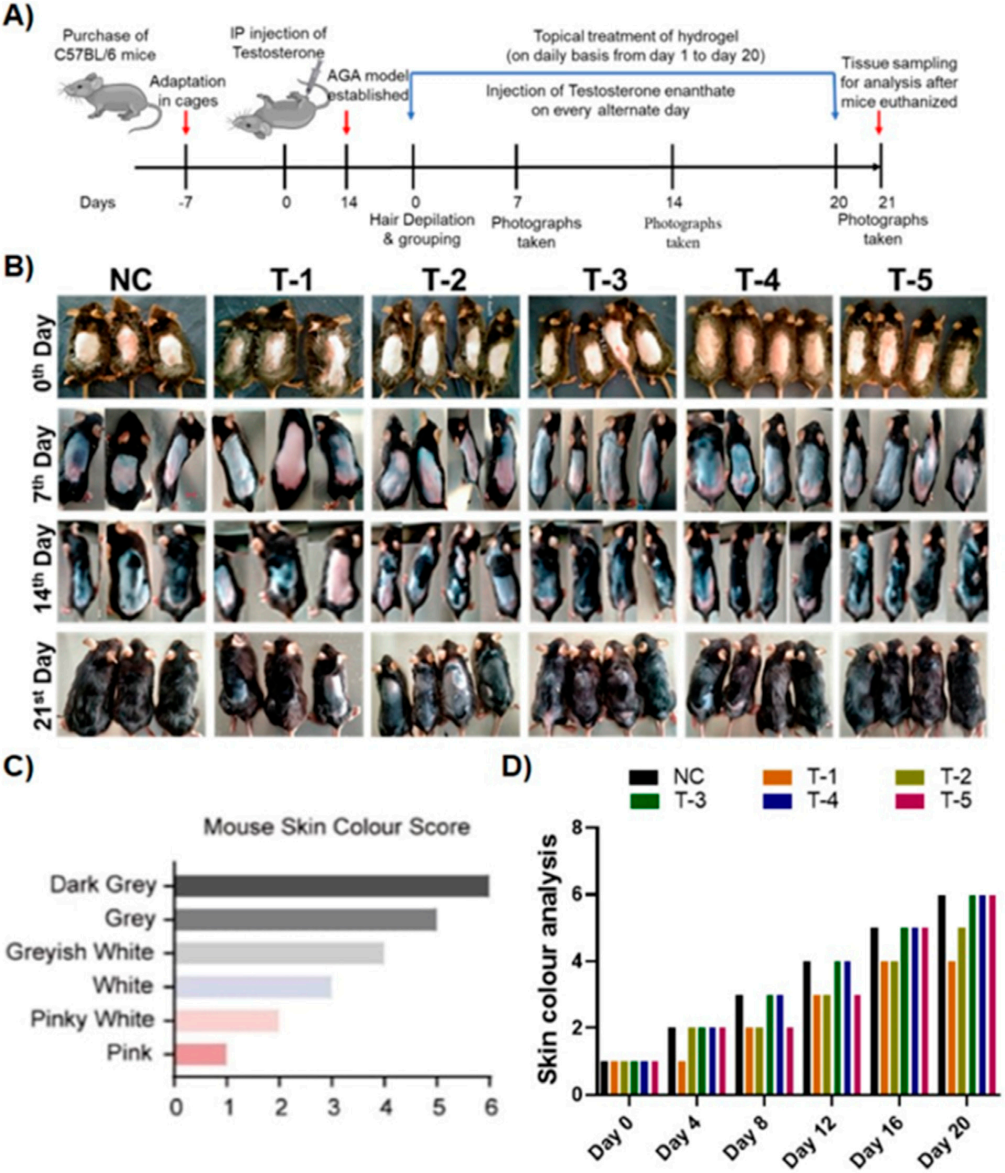
**(A)** Schematic illustration of the *in vivo* experiment. **(B)** Comparison of dorsal hair regeneration of C57BL/6 mice without any treatment (NC), testosterone (T-1), blank-SA (T-2), 2dDR-SA (T-3), minoxidil (T-4), synergistic 2dDR, and minoxidil (T-5) (*n* = 04) at different time intervals (days 0, 7, 14, and 21 of the experiment). **(C)** Mouse skin color score index. **(D)** Graphical representation of skin color scored by different treatment groups at various time intervals (days 0, 4, 8, 12, 16, and 20 of the experiment).

Hair growth was measured according to the method developed by Fu et al. (2021), by determining the skin color of mice. As melanin accumulated in the mouse skin, the color gradually changed from pink to pinkish white, white, grayish white, gray, and finally dark gray. This is because melanogenic activity of hair follicles (HFs) is tightly correlated with the hair cycle. After 7 days of full anagen phase, the skin retained a dark gray color before turning white once more due to melanocyte death, which denoted the beginning of the catagen phase. This suggests that skin color is a useful indicator of hair regrowth following depilation. The telogenic dorsal skins of C57BL/6 mice were treated topically with blank-SA and 2dDR-SA hydrogel, minoxidil 2% spray, and a combination of minoxidil 2% spray and 2dDR-hydrogel. The hydrogel was applied 5 min after minoxidil spray in the TE-induced mice in order to assess the hair regenerative activities in the AGA-induced androgenic alopecia (AGA) mouse model. It is well known that the dorsal hair of C57BL/6 mice has a time-synchronized hair growth cycle (Figure 2A). The skin color, which is bright pink during the telogen phase and turns gray or black during the anagen phase, is used as an indication that hairs are growing and is recorded in a skin color index (Figure 2C) (Van-Long et al., 2017). Until day 14, the treatment group with TE only (T-1) had extensive patches of skin lacking pigmentation, and the skin was pinkish white, scoring a 2 on the skin color score. By day 21, 30%–40% of shafts were formed in skin areas of T-1, while T-2 containing only the blank-SA hydrogel showed slightly higher skin color scores than T-1, with a skin color score of 2 on day 14 and 4 on day 21 when applied topically, however, the hair growing ability of T-3 (2dDR-SA hydrogel) in the AGA mouse model was significantly higher than that of T-1 and T-2 by day 14. The T3 group expressed a skin color score of 5 and 60%–70% of the dorsum of AGA mice was covered with hairs by day 21, and the dorsum of mice in T-3 was 90% covered with hairs, and the skin color score was 6. By comparing the T-3 group with the NC and T-4 positive control groups, no difference in the skin color score and the appearance of hair shafts at these time points were observed (Figure 2B). Additionally, mice in the T-5 group that were treated by a combination of both 2dDR-SA hydrogel and minoxidil showed white grayish skin by day 14, and hair shafts appeared at day 21. AGA mice in T-5 showed slightly delayed hair growth and change in the skin color score compared to NC, T-3, and T-4, but these parameters are still more promising than those in T-1 and T-2 (Figure 2D).

### 3.4 Hair shaft and hair length analysis

**FIGURE 3:**
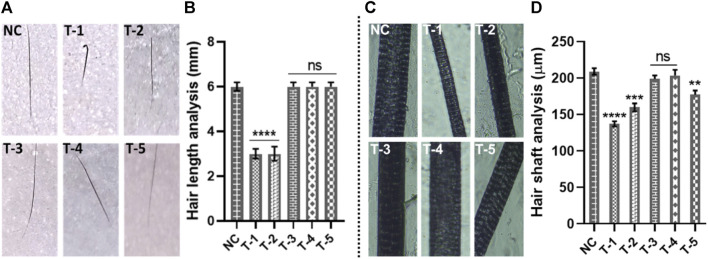
Gross analysis of hair: **(A)** digital photographs of hair length, **(B)** hair length analysis of skin sections from different treatment groups. Results are presented as *n* = 60 + SD; ****p ≤ 0.0001 denotes NC versus T-1 and T-2; ns *p* > 0.05. **(C)** Photographs of hair shafts from different treatment groups. **(D)** Hair shaft analysis of skin sections from different treatment groups. Results are presented as *n* = 60 + SD; ****p ≤ 0.0001 denotes NC versus T-1; ***p ≤ 0.001 NC versus T-2; **p ≤ 0.01 denotes NC versus T-5; and ns *p* > 0.05 denoted NC versus T-3 and T-4.

Guard, awl, auchene, and zigzag hair are four different types of hair found in mouse coats. During embryogenesis, large guard hair develops first, followed by secondary intermediates such as awl, auchene, and zigzag. Awl hair is long and straight; auchene hair has one oblique bend; and zigzag hair (shortest and most frequent) is composed of up to four segments (David et al., 2010). In this section, we only selected guard hair for measurement of the length and diameter of the hair shaft. Twenty guard hairs were plucked from the dorsum of mice, where treatments had been applied. A total of 60 guard hairs from three mice of each group were subjected to analysis. The length of guard hairs was measured by using a digital vernier caliper. The average length measured in NC was 6.04 mm, while the average length from the T-1 TE only group (2.410 mm) was lowest of all the treatment groups, and the T-3 B-SA group showed a slight increase in hair length to 2.63 mm. The average length of guard hair in T-3, T-4, and T-5 as 6.20, 6.19, and 6.02 mm, respectively (Figures 3A, B). On comparing with the normal control, no significant difference was found between T3, T4, and T5, while there was a significant difference between T1 and T2 with NC.

The second-most important factor to assess hair morphogenesis is the thickness of the hairs. We took 20 guard hairs from each mouse from the area where we had applied the treatments on the dorsum. The hair samples were observed at ×4 magnification to measure the thickness of hair shafts. The diameter of hair shafts of 20 guard hairs from all three mice from all six groups was examined. Our analysis showed the highest average hair shaft diameter in the NC group (204 µm) followed by the T-4 (201.2 µm), T-3 (198.60 µm), and T-5 (187.5 µm) groups. The lowest average hair shaft diameter (138.8 µm) was observed in T-1, without any topical treatment, and the diameter of hair shaft in the T-2 group (B-SA) was 152.4 µm. Statistical analysis showed there was no significant difference between T4 and T3, while there was a significant difference between NC and T1, T2, and T5 with the normal control. This clearly shows that 2dDR-SA has affected the morphogenesis of hair by increasing the length and thickness of mouse hair. The hair length and thickness in group T-3 (2dDR-SA) were very well-matched to those of the NC and T-4 positive control groups, which indicated that 2dDR restored the normal morphogenesis of hair by supporting the increase in hair length and thickness (Figures 3C, D).

### 3.5 Histological evaluation

After 21 days of treatment, H&E- and Masson’s trichrome-stained skin slices were microscopically analyzed, and the following factors responsible for hair growth were examined.

#### 3.5.1 Length of hair follicles

The length of the hair follicle was measured, microscopically, from horizontal sections stained with H&E skin sections. To evaluate hair follicle length, the distance between the follicular bulb residing in the dermal layer of skin and/epidermis (the upper most layer of skin) was measured. Calculations were made by counting the hair follicles per histological slide view under an inverted microscope from each of three samples in each group by keeping the scale bar at 300 μm at ×40 magnification. After day 21, the penetration of the maximum number of hair follicles into the epidermis was observed in NC, T-3, T-4, and T-5. The length (µm) of 20 hair follicles was measured by ImageJ. The average length measured in NC, T-1, T-2, T-3, T-4, and T-5 was 221 ± 0.19 µm, 134.92 ± 0.22 µm, 154.72 ± 0.32 µm, 248 ± 0.19 µm, 248 ± 0.19 µm, and 204 ± 0.29 µm, respectively. Statistically, we have found no significant difference among NC, T3, T4, and T5, while there was a significant difference among NC, T1, and T2.

**FIGURE 4:**
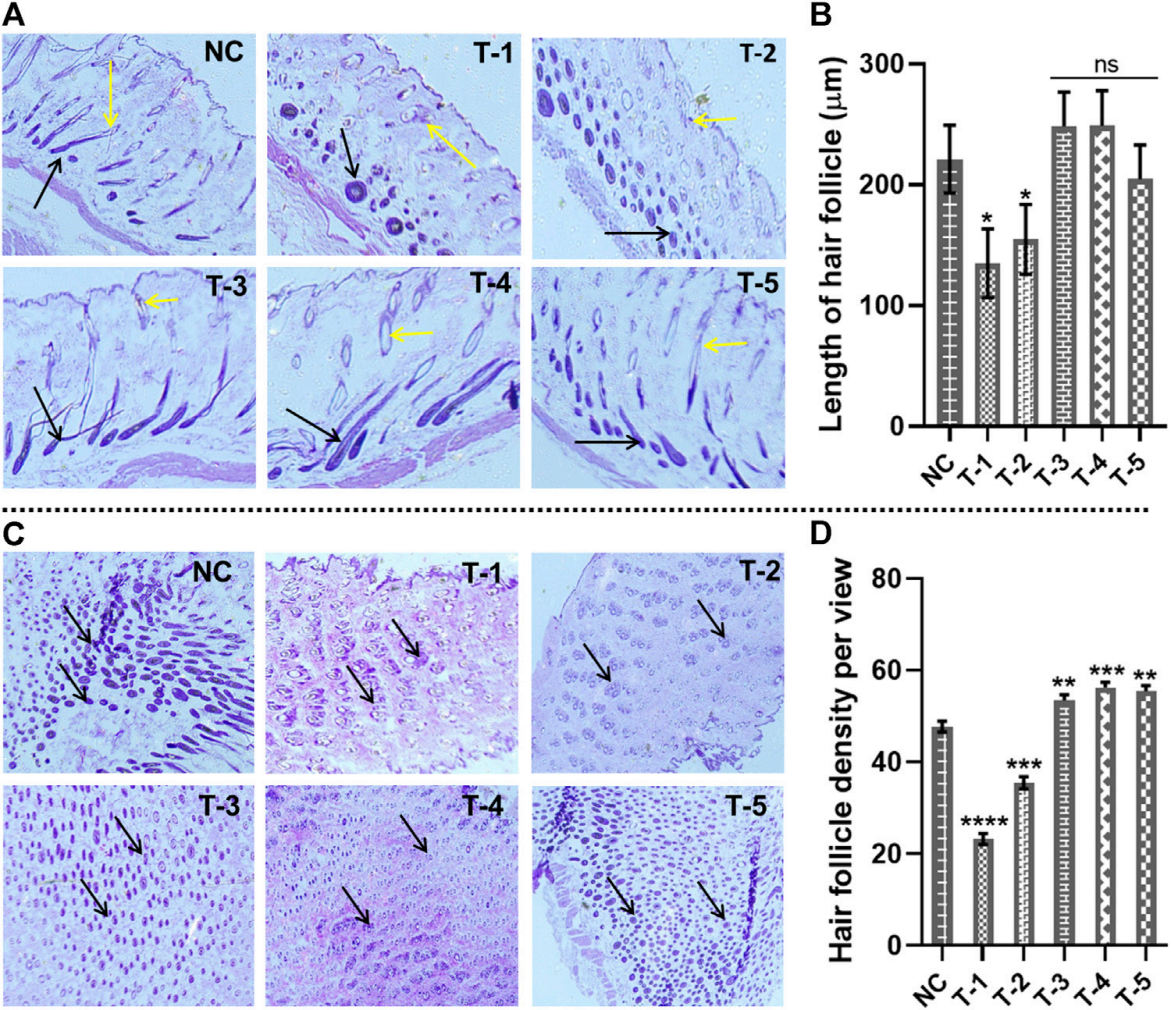
Microscopic analysis of skin sections retrieved on day 21 of experiment after H&E staining: **(A)** length of hair follicles in skin sections from different treatment groups (arrows representing the length of the hair follicles). **(B)** Comparison of the length of hair follicles in skin sections from different treatment groups. Results are presented as *n* = 4 + SD; *p ≤ 0.1 denotes NC versus T-1 and T-2; ns *p* > 0.05. **(C)** Hair follicle density in skin sections from different treatment groups (arrows representing the density of the hair follicles). **(D)** Comparison of hair follicle density in skin sections from different treatment groups. Results are presented as *n* = 4 + SD; ****p ≤ 0.0001 denotes NC versus T-1; ***p ≤ 0.001 NC versus T-2 and T-4; **p ≤ 0.01 denotes NC versus T-3 and T-5.

The histomicrographs of these two groups indicated that the hair follicles still resting in the dermis were miniaturized by the effect of AGA, while the hair follicles from T-3, T-4, and T-5 are greater in length and penetrated into the epidermis, indicating the antagonizing effects of AGA in mice (Figures 4A, B).

#### 3.5.2 Hair follicle density

To measure the hair follicle density, cross-sectional views of the skin tissue of mice stained by H&E were examined under an inverted microscope. It was observed that treatment groups T-3, T4, and T-5 had higher hair follicular densities than NC, T-1, and T-2 groups. The follicular density in the T-3, T4, and T-5 groups was recorded as 58.1, 57.6, and 56.9 hair follicles per view, while the follicular density in NC, T-1, and T-2 was 48.9, 24.34, and 38.2, respectively. The hair follicle density in T-3 (2dDR-SA hydrogel-treated group) was comparable with that of the T-4 positive control and NC group, which showed a strong hair regenerating capacity of 2dDR-SA against AGA (Figures 4C, D). There was a statistically significant difference between NC, T3, T4, and T5, with a higher hair follicle density in all these three treatment groups than in NC. The hair follicle density was significantly reduced between NC and T1 and T2.

#### 3.5.3 Diameter of the hair bulb

**FIGURE 5:**
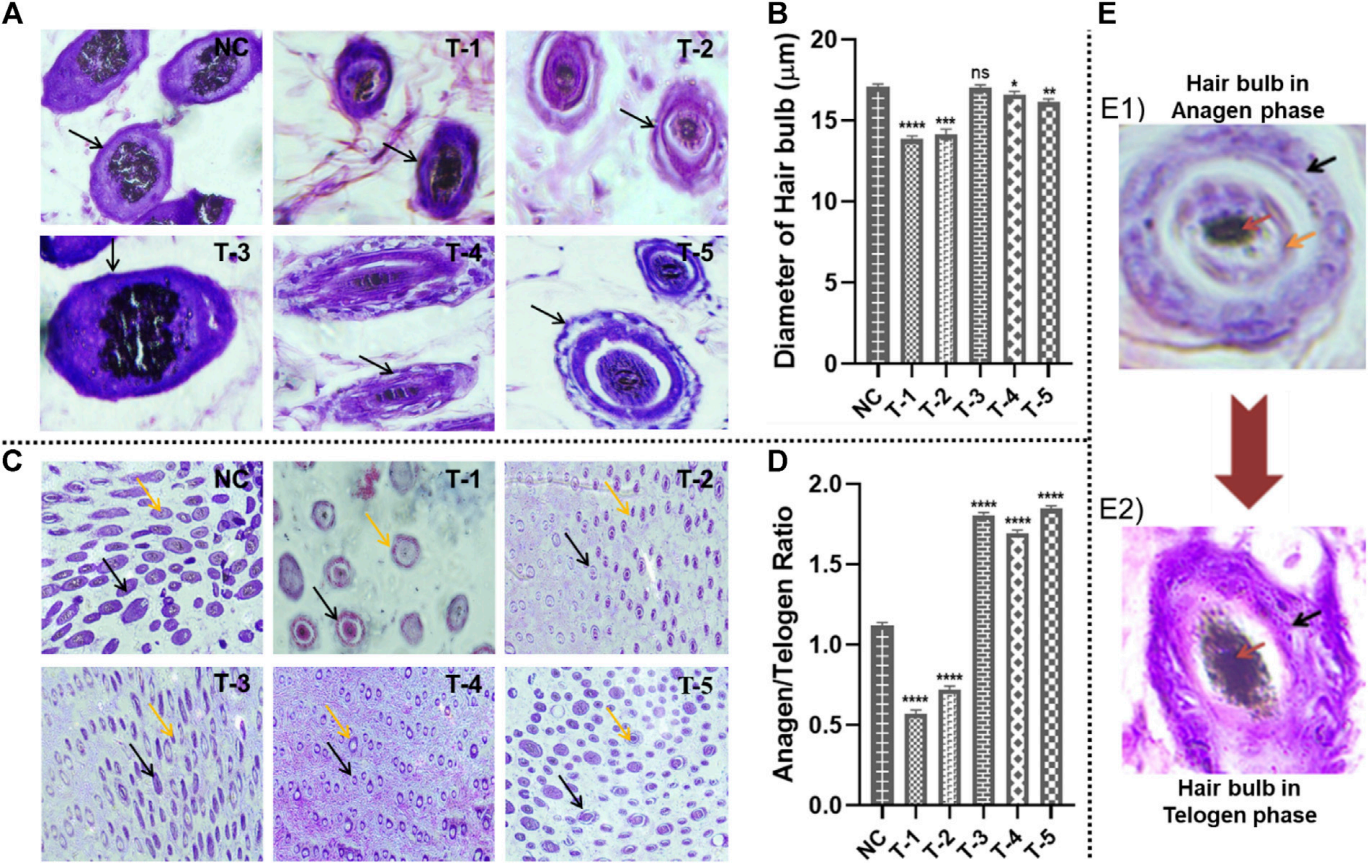
Microscopic analysis of skin sections retrieved on day 21 of experiment after H&E staining: **(A)** diameter of hair follicles in skin sections from different treatment groups (arrows representing the diameter of the hair follicles); **(B)** Comparison of the diameter of hair follicles in skin sections from different treatment groups. Results are presented as *n* = 4 + SD; ****p ≤ 0.0001 denotes NC versus T-1; ***p ≤ 0.001 NC versus T-2; **p ≤ 0.01 denotes NC versus T-5, *p ≤ 0.1 denotes NC versus T-5, and ns *p* > 0.05. **(C)** Hair follicle structures of terminal anagen and terminal telogen phases from different treatment groups (black and orange arrows representing anagen and telogen phases, respectively). **(D)** Comparison of hair follicle structures of terminal anagen and terminal telogen from different treatment groups. Results are presented as *n* = 4 + SD; ****p ≤ 0.0001 denotes NC versus T-1, T-2, T-3, T-4, and T-5. **(E)** The difference between the anagen and telogen hair bulb: **(E1)** Hair bulb in the anagen stage: black, orange, and red arrows indicating the outer root sheath, inner root sheath, and medulla, respectively, **(E2)** hair bulb in the telogen stage: black and red arrows indicating the desiccated outer root sheath and medulla, respectively, with no inner root sheath.

The diameter of the hair bulbs in all groups was measured from the cross-sections of H&E-stained skin sections of mice. For the diameter of the hair bulbs, the same trend was observed as that of hair follicle density. Diameters of the hair bulbs in the NC, T-1, T-2, T-3, T-4, and T-5 groups were calculated as 17.07 ± 0.17 μm, 13.85 ± 0.34 μm, 14.16 ± 0.17 μm, 17.52 ± 0.11 μm, 16.68 ± 0.20 μm, and 16.41 ± 0.41 μm, respectively. Among all these treatment groups, T-3 (treated with the 2dDR-SA hydrogel) exhibited the maximum diameter of bulbs in regenerated hair, as compared to treatment with minoxidil. Statistical analysis showed no significant difference between NC and T3, but there was a slight difference between NC and T4 and T5, and there was a clear significant difference between NC and T1 and T2 (Figures 5A, B).

#### 3.5.4 Anagen and telogen ratio (A/T ratio)

The A/T ratio indicates how long the anagen phase is persisting. The A/T ratio of all groups was calculated by counting the number of telogen hair follicles (T) located in the epidermis and the number of anagen hair follicles (A) located in the dermis and subcutis. On microscopic analysis, it was observed that hair follicles in the mouse skin of NC, T-1, and T-2 groups were in the epidermis and shifted earlier to the telogen phase, while T-3, T-4, and T-5 groups had follicles in the dermis and anagen phase. A/T ratios of NC, T-1, T-2, T-3, T-4, and T-5 groups were 1.2, 0.63, 0.87, 1.87, 1.69, and 1.83, respectively. This showed that hair was affected by AGA in the case of NC, T-1, and T-2, but hair regeneration was seen in T-3, T-4, and T-5 groups.

Statistical analysis showed an upward significant difference between NC, T3, T4, and T5 supporting hair follicles in the anagen phase and a downward significant difference between NC, T1, and T2. Based upon these findings, it was concluded that the 2dDR-SA hydrogel kept hair in the anagen phase (Figures 5C, D). The anagen and telogen hair follicles are identified on the basis of the presence of their outer root sheath and inner root sheath. The hair follicles in anagen show a well-defined outer and inner root sheath, while in telogen, the inner root sheath has a desiccated or wrinkled outer root sheath. Pictures of anagen and telogen phases are shown in Figure 5E.

#### 3.5.5 Area of the hair bulb covered in melanin and evaluation of blood vessels

**FIGURE 6:**
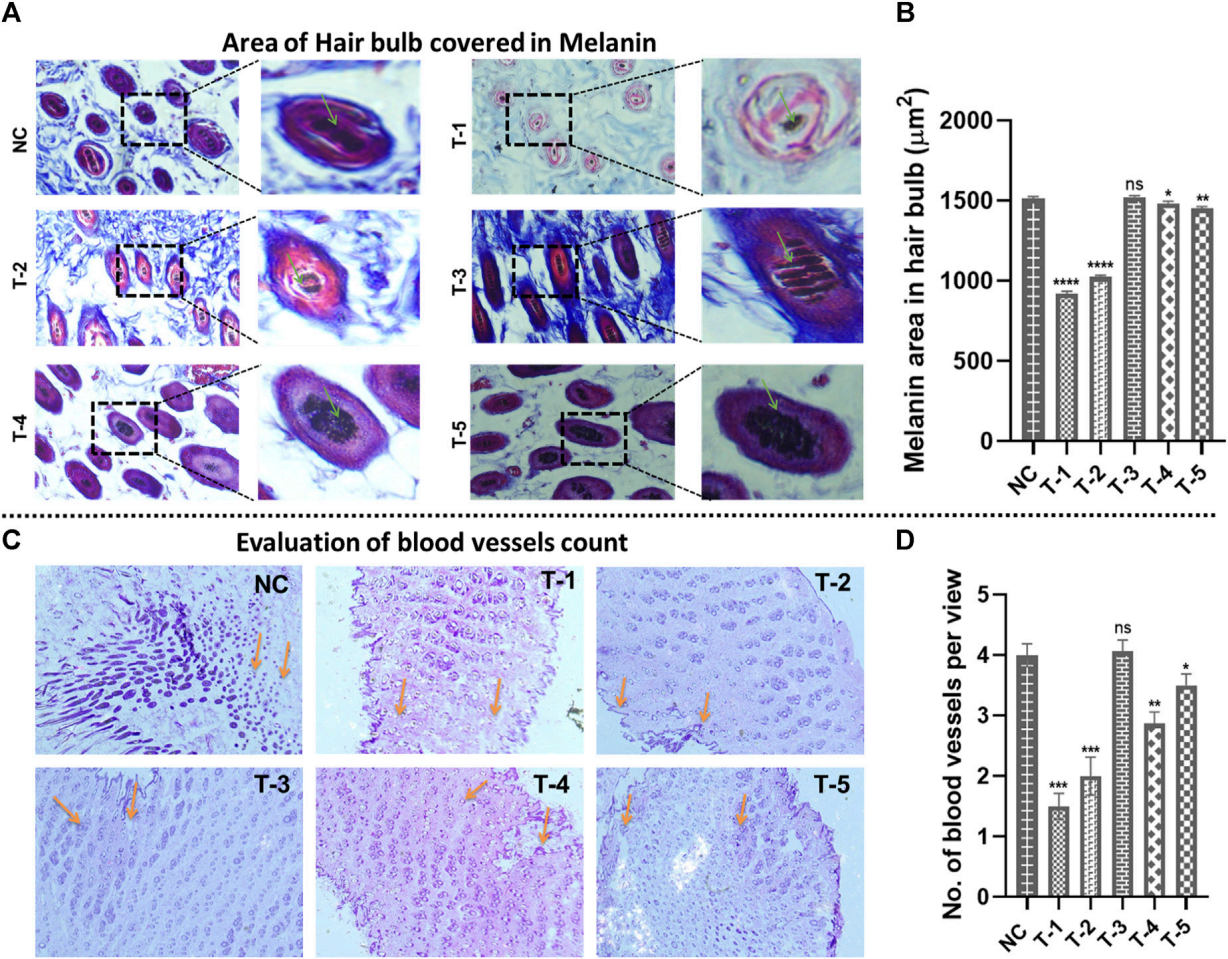
Microscopic analysis in skin sections retrieved on day 21 of experiment after H&E staining: **(A)** morphology of the area of the hair bulb covered in melanin in skin sections from different treatment groups (arrows representing the area covered by the hair bulb in melanin). **(B)** Comparison of the morphology of the area of the hair bulb covered in melanin in skin sections from different treatment groups. Results are presented as *n* = 4 + SD; ****p ≤ 0.0001 denotes NC versus T-1 and T-2; **p ≤ 0.01 denotes NC versus T-5, *p ≤ 0.1 denotes NC versus T-4, and ns *p* > 0.05 denotes NC versus T3. **(C)** The number of blood vessels from different treatment groups (arrows representing no. of blood vessels per view). **(D)** Comparison of the number of blood vessels from different treatment groups. Results are presented as *n* = 4 + SD; ***p ≤ 0.0001 denotes NC versus T-1 and T-2; **p ≤ 0.01 denotes NC versus T-4, *p ≤ 0.1 denotes NC versus T-5, and ns *p* > 0.05 denotes NC versus T3.

Area of the hair bulb covered by melanin was measured using Masson’s trichrome stained cross-sectional slices of C57BL/6 mouse skin from all groups using ImageJ software. The highest percentage of area of the hair bulb covered by melanin in hairs was found in NC, T-1, T-2, T-3, T-4, and T-5 and measured as 1,490 ± 1.29 µm^2^, 920 ± 0.32 µm^2^, 1,025 ± 0.19 µm^2^, 1,507 ± 0.917 µm^2^, 1,482 ± 1.18 µm^2^, and 1,489 ± 1.5 µm^2^, respectively. There was no statistically significant difference between NC and T3 (2dDR-SA hydrogel-treated group), but there was a significant difference between T1, T2, and NC. It was observed that there was less melanin in hair bulbs of T-1 and T-2 groups, while maximum melanin was seen in T-3, where there was hair regeneration due to the accumulation of melanin around anagenic hair bulbs (Figures 6A, B).

The effect of 2dDR on neovascularization was demonstrated by microscopic analysis of the mouse skin, and the number of blood vessels was counted using ImageJ software. The highest number of blood vessels in the dermis of the skin was found in NC (4 ± 0.191) and T-3 (4.06 ± 0.19), in the same manner as follicular density. The blood vessel count in T-1, T-2, T-4, and T-5 was 1.05 ± 0.216, 2 ± 0.32, 3.5 ± 0.21, and 3.5 ± 0.26, respectively. In summary, angiogenesis was reduced in the DHT model, but significantly increased in 2dDR and minoxidil models (Figures 6C, D).

## 4 Discussion

In our first study of the effects of 2dDR on wound healing in animals (Serkan et al., 2021), we observed apparent stimulation of the hair follicles adjacent to the wound. We did not investigate this further at that time, but now, we critically observed the effect of 2dDR on hair regrowth. To do this, we used a well-established mouse model of androgen-induced hair loss. This study shows a positive effect of 2dDR on hair regrowth in this animal model using the alginate hydrogel as a carrier to deliver 2dDR in a sustained manner.

Blank-SA and 2dDR-SA hydrogels were prepared by simple manual mixing of alginate, propylene glycol, phenol ethanol, and 2dDR in water. These were characterized by FTIR, which confirmed no evidence of chemical changes in gels on the addition of 2dDR. *In vitro* drug release showed sustained release of 2dDR for 7 days.

Male pattern baldness, or androgenic alopecia, is the most common type of hair loss worldwide (María et al., 2018). It is caused by abnormal androgen expression and metabolic effects, with key components being dihydrotestosterone (DHT) and 5α-reductase. The DHT-AR complex triggers TGF-2, inhibiting cell proliferation, leading to early hair follicle entry into the catagen phase (Francesca et al., 2017).

The treatment of AGA remains challenging, with only two FDA-approved drugs being used, minoxidil and finasteride (Maurizio et al., 2016). Finasteride and minoxidil stimulate hair growth, but both have some negative effects.

Angiogenesis is known to help hair regrowth, and our previous studies showed the ability of 2dDR to promote angiogenesis (Serkan et al., 2019). In our current research, we tested the effect of 2dDR on AGA in a mouse model. For the topical delivery of 2dDR, the alginate gel was used as a carrier. This alginate-based 2dDR-SA hydrogel showed sustained release of 2dDR.

To develop the AGA model in C57BL/6 mice, we injected testosterone intraperitoneally three times a week. We found that the 2dDR-SA hydrogel-treated mice showed a similar effect of hair regrowth as the positive control group (minoxidil) by 21 days. We quantified the hair regrowth activity by investigating gross morphogenesis parameters such as a change in skin color, hair length, and hair shaft diameter. The 2dDR-SA hydrogel supported hair regrowth.

Anagen, catagen, and telogen are the three stages of hair follicle growth. The anagen phase is characterized by an increase in follicle length, robust hair growth, and thickening of the hair shaft. In the telogen and catagen phases, the length of the follicle decreases, the hair shaft becomes thinner and wrinkled, and hair loss develops (Sven et al., 2001). The more hair follicles in the anagen phase, the better the hair growth. We noted that 2dDR-SA hydrogel-treated mice showed a high anagen ratio compared to the negative control group and blank-SA hydrogel group.

Our histological investigations demonstrated that 2dDR-SA hydrogel increased hair development by elongating the anagen phase, which was shortened in AGA. To thoroughly evaluate the hair regeneration capacity of the 2dDR-SA hydrogel, six parameters were selected: length of hair follicles, density of hair follicles, A/T ratio, diameter of hair follicles, area of the hair bulb covered in melanin, and the number of blood vessels. In the 2dDR-SA group, a significant increase in hair follicle length and dense hair follicles were observed, similar to the positive control group (minoxidil)-treated group. The dermal papilla cells (DPCs) in the hair bulbs are responsible for follicle growth. The growth phase of the hair follicle is determined by the rate of dermal papilla cell proliferation, which helps maintain the diameter of the hair bulb and promotes hair growth in the anagen phase. The hair bulb is smallest in size when the follicles are in the telogen phase, where the DPCs are dormant. In the present study, the mice treated with the 2dDR-SA hydrogel had hair bulb diameters like those in the normal controls and greater hair follicle density compared to the negative control and blank-SA hydrogel-treated groups.

The stimulation of angiogenesis around the hair bulb can control its size. The better the blood supply to the hair bulb, the larger its diameter and the more hair growth (Supino, 1995). We counted the blood vessels in the horizontal sections of H&E-stained tissues using an inverted microscope. 2dDR-SA hydrogel and minoxidil-treated groups showed an increase in the number of blood vessels compared to the group treated with blank-SA hydrogel. Blood vessels were slightly greater in number in the mice treated with 2dDR-SA.

The synthesis of stem cell factor in DPCs has been known to be inhibited by testosterone, which prevents bulbar melanocyte pigmentation and results in pallor in hair of AGA patients. In the present study, the mouse skin samples were stained with Masson’s trichrome and examined micromorphologically and photographed using an inverted microscope. 2dDR-SA hydrogel treatment increased melanin synthesis in the hair bulbs of the C57BL/6 mouse model. Overall, 2dDR-SA hydrogel stimulated hair growth in the AGA mouse model, and its effect was similar to that of minoxidil, an FDA-approved drug.

Finally, as the positive effects of 2dDR were possibly associated with a stimulation of angiogenesis, it would work on other causes of hair loss such as chemotherapy-induced alopecia (María et al., 2018). This is responsible for low self-esteem, anxiety, depression, poor body image, and disturbed thinking (Erol et al., 2012; Jayde et al., 2013). A bald head and loss of eyebrows and eye lashes can result from cancer treatment, and this can put patients under great social distress. This is a badly under-researched area, and hence new approaches are needed. One example of new thinking in the area is a recent pivotal study which shows how paradoxically inhibiting proliferation of hair bulb cells prior to treatment with chemotherapeutic agents protects these cells from the most damaging effect of chemotherapeutic agents (Purba et al., 2019).

## 5 Conclusion

The study showed the effectiveness of the 2dDR-SA hydrogel in stimulating hair regrowth in an animal model of male androgen-induced hair loss. Parameters such as the length of hair, hair shaft diameter, length of hair follicles, density of hair follicles, A/T ratio, diameter of hair follicles, area of hair bulbs covered in melanin, and blood vessel count all confirmed effective hair regrowth after the application of the 2dDR-SA hydrogel. This was found possibly to be as effective as the FDA-approved drug minoxidil.

In conclusion, this manuscript demonstrates that 2dDR stimulates hair growth in an animal model of androgenic alopecia. As such, it is the first study to do so. Our tentative hypothesis is that 2dDR upregulates VEGF in this animal model, leading in turn to the stimulation of angiogenesis and stimulation of new hair growth. However, to study the mechanism of action of 2dDR, further work will be required to investigate the levels of VEGF in this model, following the addition of 2dDR, and to what extent the hair follicle stimulation can be blocked by the addition of VEGF inhibitors.

## Data Availability

The original contributions presented in the study are included in the article/Supplementary Material; further inquiries can be directed to the corresponding authors.
