# Corrigendum: Stimulation of hair regrowth in an animal model of androgenic alopecia using 2-deoxy-D-ribose

**DOI:** 10.3389/fphar.2024.1499205

**Published:** 2024-10-25

**Authors:** Muhammad Awais Anjum, Saima Zulfiqar, Aqif Anwar Chaudhary, Itesham Ur Rehman, Anthony J. Bullock, Muhammad Yar, Sheila MacNeil

**Affiliations:** ^1^ Interdisciplinary Research Center in Biomedical Materials, COMSATS University Islamabad, Lahore Campus, Lahore, Pakistan; ^2^ School of Medicine, University of Central Lancashire, Preston, United Kingdom; ^3^ Department of Materials Science and Engineering, Kroto Research Institute, University of Sheffield, Sheffield, United Kingdom

**Keywords:** androgenic alopecia, 2-deoxy-D-ribose, C57BL6 mice, testosterone, minoxidil, hair regrowth, chemotherapy

In the published article, there was an error in Figure 2 as published. The data graph 2D representing skin color scores had erroneous error bars applied. The corrected Figure 2 and its caption appear below.

**FIGURE 2:**
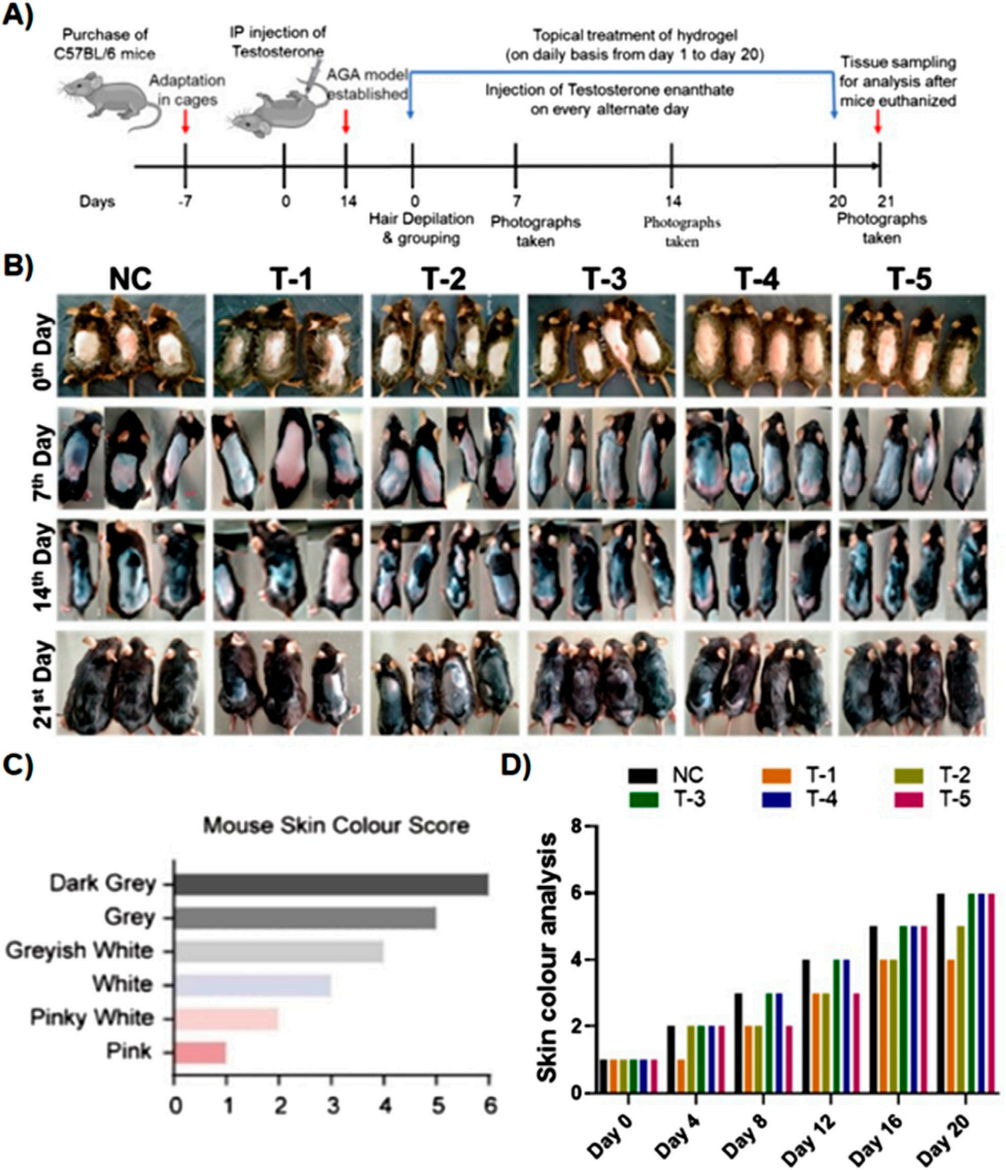
**(A)** Schematic illustration of the *in vivo* experiment. **(B)** Comparison of dorsal hair regeneration of C57BL/6 mice without any treatment (NC), testosterone (T-1), blank-SA (T-2), 2dDR-SA (T-3), minoxidil (T-4), synergistic 2dDR, and minoxidil (T-5) (n = 04) at different time intervals (days 0, 7, 14, and 21 of the experiment). **(C)** Mouse skin color score index. **(D)** Graphical representation of skin color scored by different treatment groups at various time intervals (days 0, 4, 8, 12, 16, and 20 of the experiment).

The authors apologize for this error and state that this does not change the scientific conclusions of the article in any way. The original article has been updated.

